# Impact of physical activity on cognitive function in long COVID patients: a cross-sectional observational study

**DOI:** 10.3389/fspor.2026.1738512

**Published:** 2026-05-07

**Authors:** Samuel da Conceição Dummer, João Ismael Budelon Gonçalves, Fábio Jean Varella, Mariana Rache Zamin, Guilherme Kripka, Frederico Friedrich, Marcus Herbert Jones, Gabriela de Oliveira Centofante, Fernanda Noschang da Rocha Colcete, Jaderson Costa Da Costa, Daniel Rodrigo Marinowic

**Affiliations:** 1 Brain Institute of Rio Grande do Sul (BraIns), Pontifical Catholic University of Rio Grande do Sul, Porto Alegre, Brazil; 2 Graduate Program in Medicine, Pediatrics and Child Health, Medical School, Pontifical Catholic University of Rio Grande do Sul, Porto Alegre, Brazil; 3 Graduate Program in Medicine and Health Sciences, Medical School, Pontifical Catholic University of Rio Grande do Sul, Porto Alegre, Brazil; 4 Centro Infant, Department of Pediatrics, School of Medicine, Pontifical Catholic University of Rio Grande do Sul (PUCRS), Porto Alegre, Brazil

**Keywords:** cognitive function, long covid, physical fitness, post-COVID-19 abnormalities, VO_2_ max

## Abstract

**Introduction:**

The COVID-19 pandemic has resulted in long-term sequelae known as long COVID, which is often characterized by cognitive dysfunctions that impair quality of life. Evidence suggests that physical activity may mitigate these impairments through neurobiological mechanisms that enhance neuroplasticity and cerebral perfusion.

**Objective:**

This study examined the association between physical fitness and cognitive function in individuals who had recovered from COVID-19.

**Methods:**

A cross-sectional observational study was conducted involving 34 adults who had been previously hospitalized for COVID-19. Cognitive performance was assessed using the Addenbrooke's Cognitive Examination–Revised (ACE-R), while cardiorespiratory fitness was evaluated through VO₂ max testing. Correlation analyses and generalized linear models adjusted for age, sex, and body mass index were applied to examine associations between physical fitness and cognitive domains.

**Results:**

Participants had a mean age of 52 ± 12 years, a BMI of 28.7 ± 4.4 kg/m^2^, and a mean VO₂ max of 36 ± 9 mL/kg·min⁻^1^. A strong positive correlation was observed between VO₂ max and the total ACE-R score (*r* = 0.653; *p* < 0.001), with the memory domain showing the strongest association (*r* = 0.739; *p* < 0.001). Higher physical activity levels, as assessed by the IPAQ, were also associated with better cognitive outcomes.

**Discussion:**

Physical fitness was significantly associated with better cognitive performance in post-COVID-19 patients. These findings support the inclusion of structured exercise programs in rehabilitation strategies to mitigate the cognitive sequelae of long COVID and underscore the importance of promoting physical activity as a public health intervention.

## 1 Introduction

The SARS-CoV-2 pandemic had profound global health impacts, leading the WHO to declare it a pandemic in March 2020. Measures such as social distancing helped mitigate the spread of the virus but also worsened mental health conditions, including depression and anxiety. Furthermore, many individuals continue to experience post-COVID-19 syndrome, or long COVID, characterized by persistent symptoms such as fatigue and cognitive dysfunction, which significantly impair quality of life (1–4).

Given the significant impact of COVID-19 on global health, it is imperative to investigate associations between factors and outcomes related to the persistent symptoms of long COVID, in order to deepen the understanding of the condition and develop targeted interventions. Cognitive dysfunction, in particular, has emerged as a major concern, affecting memory, attention, and executive functions in millions of individuals (5). The World Health Organization has defined this condition as “post-COVID-19 syndrome,” referring to a wide range of persistent or newly developed symptoms of a continuous or fluctuating nature that occur 12 weeks after a microbiologically confirmed or suspected SARS-CoV-2 infection and cannot be explained by an alternative diagnosis (6). Regular physical exercise, particularly in the context of the COVID-19 pandemic, is a well-established strategy known to benefit both the immune system and cognitive health, and has emerged as a potential intervention to mitigate these deficits (7, 8).

Studies indicate that physical exercise can enhance the production of neurotrophic factors, promote neuroplasticity, and improve neural connectivity, suggesting that physical activity may serve as an effective non-pharmacological intervention to alleviate cognitive deficits associated with COVID-19 (9–11). Supervised exercise programs have also been shown to reduce fatigue and enhance quality of life in patients with long COVID, underscoring the importance of further investigating effective interventions to address the long-term effects of COVID-19.

Mechanistic evidence suggests that post-COVID cognitive dysfunction involves processes such as persistent neuroinflammation and alterations in cerebral perfusion, potentially compounded by endothelial dysfunction; these mechanisms are associated with difficulties in memory, attention, and processing speed observed among COVID-19 survivors. In parallel, clinical studies have documented measurable cognitive impairments in patient samples following infection, reinforcing the clinical relevance of this issue (12, 13).

In this context, physical activity may exert multisystem effects—modulating inflammatory, vascular, and neuroplastic pathways—and is associated with improved functional outcomes in long COVID. Recent reviews indicate the benefits of exercise-based rehabilitation programs and suggest that higher levels of physical activity are linked to a lower symptom burden over prolonged follow-up, underscoring the importance of investigating, with physiological precision (e.g., VO₂ max), the relationship between cardiorespiratory fitness and cognitive performance in COVID-19 survivors (14, 15).

Considering the long-term effects of COVID-19, particularly on mental health and its potential relationship with physical activity levels, it is essential to expand our understanding of this emerging condition. Studies indicate that individuals with long COVID experience reduced exercise capacity and cognitive impairments, affecting memory, attention, and processing speed (16). Conversely, regular physical activity before and during the pandemic has been associated with fewer sequelae and faster recovery, suggesting a protective effect of physical fitness on post-COVID outcomes (17). In this context, we examined the relation between physical fitness and cognitive function in patients who had recovered from COVID-19.

## 2 Methods

### 2.1 Ethical aspects

This study was approved by the Research Ethics Committee of the Pontifical Catholic University of Rio Grande do Sul (CAAE: 30784620.3.0000.5336, approval number: 6.123.125). All participants provided written informed consent, in accordance with Resolution No. 466/2012 of the Brazilian National Health Council.

### 2.2 Participant description

**Figure 1:**
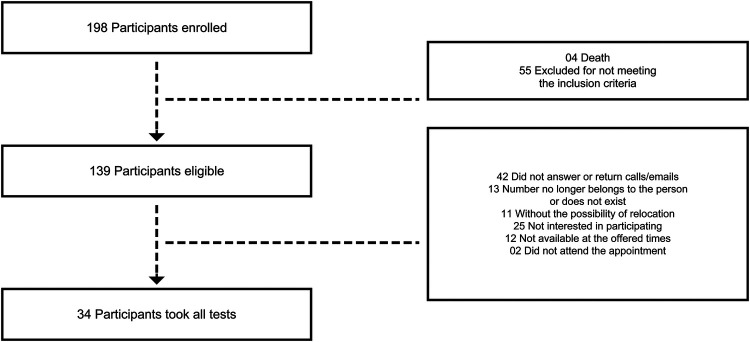
Diagram illustrating the flow of participant enrollment in the study. The inclusion and exclusion criteria were applied, along with consideration of participants' right to withdraw or potential difficulties encountered during the study.

Patients who were admitted to São Lucas Hospital at PUCRS for COVID-19 symptoms in 2021 and tested positive for SARS-CoV-2 via oropharyngeal swab PCR were included in the study. All participants were literate and aged between 18 and 80 years. Individuals with severe medical conditions that precluded physical exercise or with diseases potentially impairing memory and cognition were excluded. The thirty-four adult participants were recruited for this study two years and seven months after COVID-19 infection and hospitalization. Most participant exclusions occurred because individuals did not meet the inclusion criteria, which were considered essential for the application of all study assessments. Subsequently, exclusions were due to lack of response to telephone calls and emails sent by the research team, lack of interest in participating in the study, changes in phone numbers during the recruitment period, unavailability of time or transportation to participate, and, finally, withdrawal during the recruitment process. The recruitment and data collection process is illustrated in Figure 1.

### 2.3 Clinical data of hospitalization

Specific information was collected for participants who required admission to a general ward or an intensive care unit (ICU), including the degree of pulmonary involvement on chest computed tomography (CT) and the comorbidity index (Charlson Score). In addition, participants provided their past medical history, current symptoms, and symptoms since the onset of infection, as well as information regarding hospitalization history. All participants presented at least some persistent symptoms weeks or months after SARS-CoV-2 infection. Among the reported symptoms, the most prominent were respiratory difficulty, cough, fatigue, mental health problems, behavioral changes, and cognitive difficulties, meeting the definition of long COVID.

### 2.4 Cognitive function assessment

Participants underwent the Addenbrooke's Cognitive Examination–Revised (ACE-R) at the Brain Institute of Rio Grande do Sul (BraIns)–PUCRS over a two-month period. The test assessed five cognitive domains: attention and orientation, memory, verbal fluency, language, and visuospatial abilities. In addition, symptoms of depression and anxiety, as well as participants' levels of resilience, were assessed. The evaluation was performed using the Hamilton Scale, consisting of 21 items for depression and 14 items for anxiety.

### 2.5 Ergospirometry

For the assessment of physical effort, participants were taken to the Movement Center of PUCRS, where an ergometric protocol was conducted, starting at a speed of 4 km/h and progressively increasing until voluntary exhaustion. Heart rate (HR) was monitored using an electrocardiogram and Mortara® software, both connected to the examination computer. Data were exported and stored in the system's cloud. Ventilatory thresholds and VO₂ max were determined by an experienced physiologist. Notably, all evaluating professionals were blinded to the results of other tests, and each assessment was administered by different professionals, ensuring the impartiality and validity of the collected data.

### 2.6 Statistical analysis

Statistical analyses were conducted using R software (version 4.2.0), a robust and widely used environment for statistical computing and graphics. The analysis script is publicly available in Online Resource 4. To explore the relationship between cognitive domain scores, assessed by the ACE-R, and cardiorespiratory fitness, measured by VO₂ max, Pearson correlation analyses were performed. In addition to descriptive and correlation analyses, generalized linear models (GLMs) (Online Resource 2) were employed to assess the influence of additional covariates on the results. These analyses controlled for potential confounding factors such as age, sex, and body mass index (BMI), educational level, disease severity, and comorbidities, providing a more detailed understanding of the relationship between physical activity levels, cardiorespiratory fitness, and cognitive domain scores. The GLM results reinforced the significance of the observed associations after adjusting for the effects of the covariates included in the model. Statistical significance was set at *p* < 0.05 for all analyses.

## 3 Results

In this study, a total of 34 volunteer participants were enrolled and completed all stages of the investigation. Of the participants, 53% were male and 47% were female, with a mean age of 52 ± 12 years. Evaluation of the Charlson Comorbidity Index classified 58.33% of participants as low risk, 29.17% as intermediate risk, and 12.5% as high risk. Based on radiological assessment, 62.5% of participants exhibited mild pulmonary involvement on chest computed tomography (CT), while 37.5% showed severe involvement.

The mean weight was 82.8 ± 17.4 kg, height was 1.69 ± 0.08 m, and body mass index (BMI) was 28.7 ± 4.4 kg/m^2^. The average VO₂ max was 36 ± 9 mL/kg·min⁻^1^. The mean length of hospital stay was 7.8 ± 6.4 days (IQR 3.3–10.0 days), and only 5.9% of participants (2/34) required admission to the intensive care unit (ICU).

In the Addenbrooke's Cognitive Examination–Revised (ACE-R), the mean domain scores were: attention and orientation, 16.4 (SD = 1.04); memory, 18.56 (SD = 3.94); verbal fluency, 10.94 (SD = 2.21); language, 24.74 (SD = 2.02); and visuospatial function, 14.06 (SD = 2.23). Demographic data and additional test results are presented in Table 1.

**Table 1:** Demographic, clinical, and cognitive characteristics of the participants.

Variables	Enrolled patients (*N* =34)
Gender, *n* (%)	
Male	18 (52.9)
Female	16 (47.1)
Age, years (Mea*n* ± SD)	52 ± 12
Weight, (kg), (Mean ± SD)	82.8 ± 17.4
Height, (m), (Mean ± SD)	1.69 ± 0.08
Charlson Comorbidity Index, *n* (%)	
Low risk	20 (58.3)
Intermediate risk	10 (29.2)
High risk	4 (12.5)
BMI, kg/m^2^, (Mean ± SD)	28.7 ± 4.4
VO₂max, mL/kg.min^−1^, (Mean ± SD)	36 ± 9
Chest CT, *n* (%)	
Mild pulmonary involvement	21 (62.5)
Severe pulmonary involvement	13 (37.5)
Hospital length of stay, days (Mean ± SD)	7.8 ± 6.4
IPAQ questionnaire scores, *n* (%)	
Sedentary	7 (20.6)
Insufficiently active	12 (35.3)
Active	11 (32.3)
Very active	4 (11.8)
ACE—Attention and Orientation, (Mean ± SD)	16.4 ± 1.04
ACE—Memory, (Mean ± SD)	18.56 ± 3.94
ACE—Verbal Fluency, (Mean ± SD)	10.94 ± 2.21
ACE—Language, (Mean ± SD)	24.74 ± 2.02
ACE—Visuospatial Function, (Mean ± SD)	14.06 ± 2.23

Data are presented as *n* (%) and mean (SD). ACE-R, addenbrooke's cognitive examination-revised; BMI, body-mass index; IPAQ, international physical activity questionnaire; CT, computed tomography; VO_2_max, maximal oxygen uptake.

**Figure 2:**
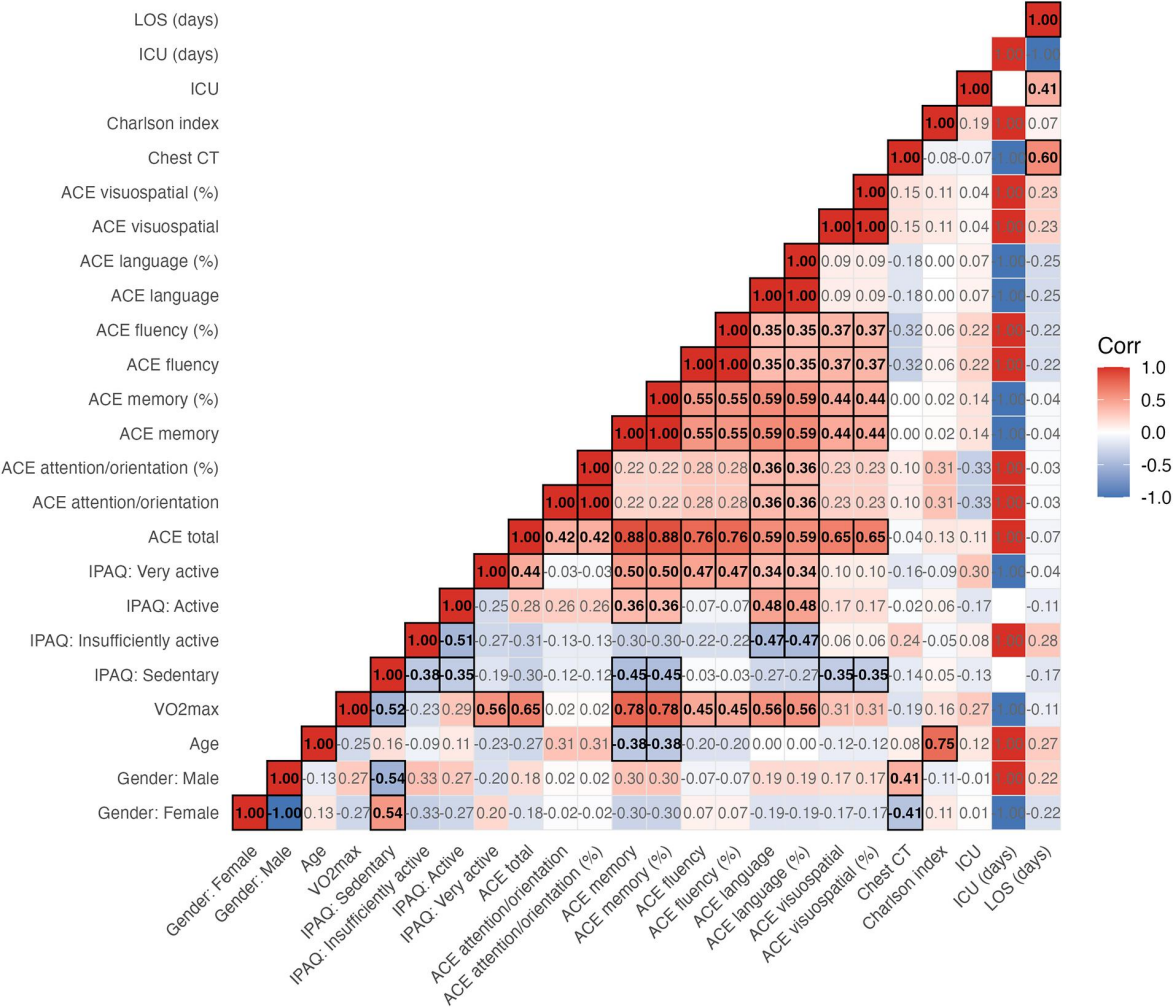
Correlation matrix of all analyzed parameters. Sociodemographic variables, VO₂, IPAQ scores, cognitive scores, and clinical variables—including disease severity, pulmonary involvement on chest computed tomography (CT), comorbidity index (Charlson Score), and outcomes—were analyzed to identify statistically significant correlation coefficients.

A Spearman correlation analysis was performed between the selected quantitative and qualitative variables as an exploratory assessment, aiming to investigate potential monotonic associations among demographic data, physical fitness (VO_2_), IPAQ questionnaire scores, cognitive scores, and clinical variables, including disease severity, pulmonary involvement on chest computed tomography (CT), comorbidity index (Charlson Score), and outcomes (Figure 2). The correlation values between VO_2_max, IPAQ, and ACE-R cognitive domains are described in Table 2.

**Table 2:** Key correlations between VO_2_max, IPAQ, and ACE-R cognitive domains.

Predictor Variable	Cognitive Outcome (ACE-R)	Coefficient (r)	*p*-value
VO_2_max	Total score	0.65	<0.01
VO_2_max	Memory	0.78	<0.01
VO_2_max	Language	0.56	<0.01
IPAQ—Very Active	Total score	0.44	<0.01
IPAQ—Very Active	Memory	0.50	<0.01
IPAQ—Very Active	Language	0.48	<0.01
IPAQ—Active	Memory	0.36	<0.01
IPAQ—Active	Language	0.34	<0.01

ACE-R, addenbrooke's cognitive examination-revised; IPAQ, international physical activity questionnaire; VO_2_max, maximal oxygen uptake.

For the total Addenbrooke's Examination score, the correlation coefficient was 0.56 (*p* < 0.01), indicating a moderate and statistically significant association. Higher physical activity levels, as measured by the IPAQ, were associated with better overall performance on the Addenbrooke's. Specifically, being classified as “Active” was associated with an 8.58-point increase in the total score (*p* = 0.019), while being “Very Active” was associated with a 14.36-point increase (*p* = 0.003). Conversely, being “Insufficiently Active” showed no significant association (Estimate = 1.77, *p* = 0.61).

For the Memory domain, the correlation coefficient was 0.63 (*p* < 0.01), indicating a strong and statistically significant relationship. Higher physical activity levels were associated with significant improvements in the Memory domain of the Addenbrooke's Examination. Specifically, being classified as “Active” was associated with a 16.79-point increase in the Memory domain (95% CI: 4.90–28.67, *p* < 0.01), while being “Very Active” was associated with a 28.85-point increase (95% CI: 13.44–44.26, *p* < 0.01). In contrast, being “Insufficiently Active” did not show a significant association (Estimate = 2.89, 95% CI: −8.80–14.58, *p* = 0.62).

For the Language domain, the correlation coefficient was 0.48 (*p* < 0.01), indicating a moderate and statistically significant relationship. Higher physical activity levels, as measured by the IPAQ, were associated with significant improvements in the Language domain of the Addenbrooke's Examination. Specifically, being classified as “Active” was associated with an 8.64-point increase in the Language domain (*p* = 0.016), while being “Very Active” was associated with a 9.34-point increase (*p* = 0.041). In contrast, being “Insufficiently Active” did not show a significant association (Estimate = 1.65, *p* = 0.62).

**Figure 3:**
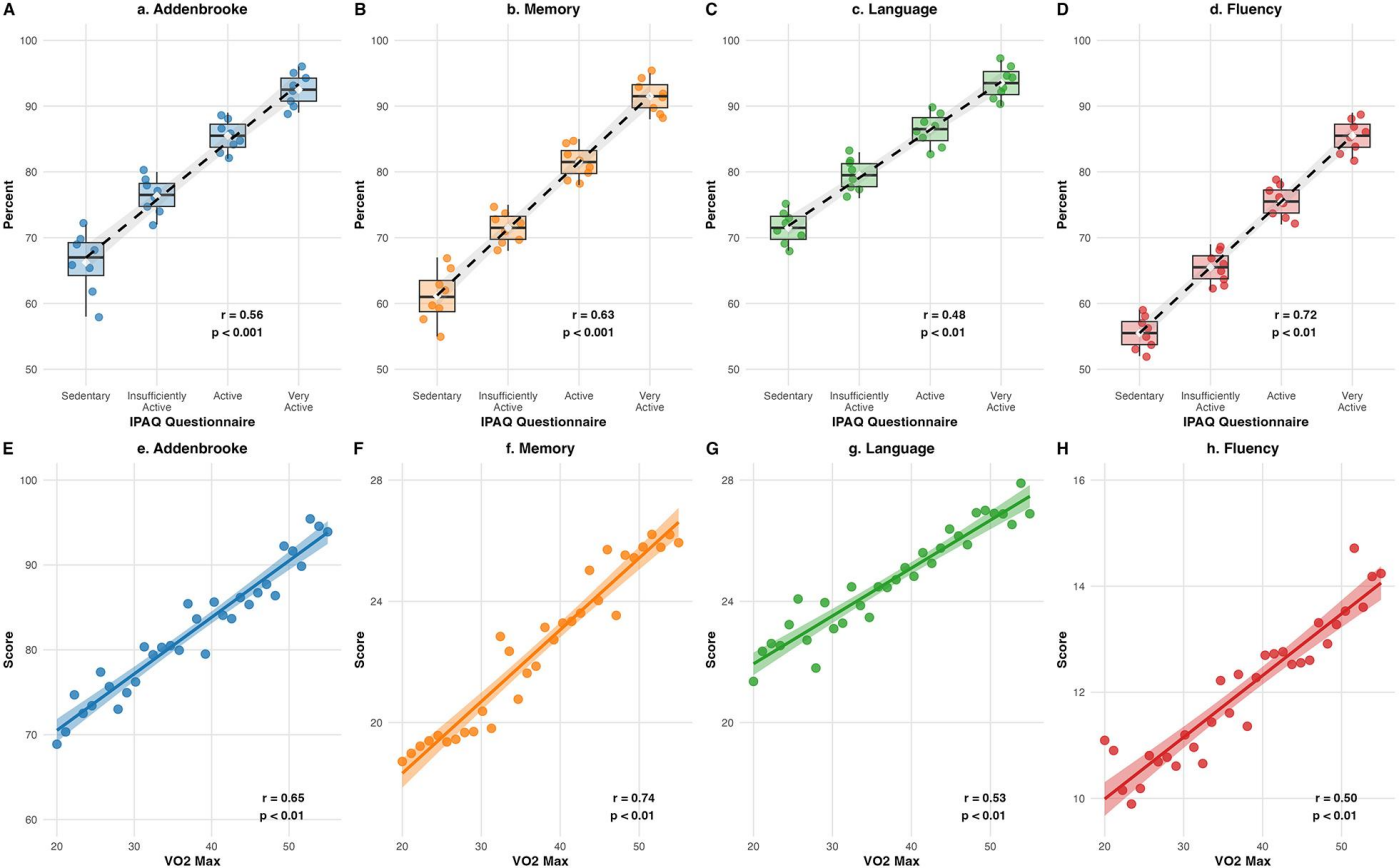
Cognitive assessment profiles across physical activity and fitness measures. Upper panels: Frequency distributions of ACE-R cognitive domains correlated with IPAQ questionnaire scores, including Addenbrooke's total score **(A)**, Memory **(B)**, Language **(C)**, and Fluency **(D)**. Lower panels: Frequency distributions of ACE-R cognitive domains correlated with VO_2_max levels, including Addenbrooke's total score **(E)**, Memory **(F)**, Language **(G)**, and Fluency **(H)**.

The correlation between the overall ACE-R cognitive domain scores and VO₂ max was 0.653 (*p* < 0.001), indicating a strong overall association (Figure 3). A positive correlation was observed between the ACE-R memory score (%) and VO₂ max, with a correlation coefficient of 0.739 (*p* < 0.001), demonstrating a substantial and statistically significant relationship. VO₂ max was significantly associated with multiple cognitive performance measures assessed by the ACE-R. Specifically, VO₂ max showed a positive association with memory (coefficient = 1.21; 95% CI = 0.71–1.72; *p* < 0.001), overall score (coefficient = 0.69; 95% CI = 0.35–1.03; *p* < 0.001), language (coefficient = 0.48; 95% CI = 0.16–0.81; *p* = 0.007), and verbal fluency (coefficient = 0.86; 95% CI = 0.32–1.39; *p* = 0.002). Additionally, length of hospital stay (LOS) was negatively associated with language performance (coefficient = −0.81; 95% CI = −1.48 to −0.14; *p* = 0.025). Age was positively associated with attention (coefficient = 0.22; 95% CI = 0.02–0.41; *p* = 0.036), and male sex was positively associated with language performance (coefficient = 7.43; 95% CI = 0.46–1.43; *p* = 0.047).

## 4 Discussion

In this study, we evaluated 34 participants who had been hospitalized for COVID-19 to investigate the relationship between physical fitness (IPAQ and VO₂ max) and cognitive function (ACE-R). We observed a significant positive correlation between physical fitness and various measures of cognitive performance, including memory, language, and the overall ACE-R score. Our findings have important clinical implications and are consistent with existing literature supporting the cognitive benefits of physical exercise. Previous studies suggest that physical exercise can enhance the production of neurotrophic factors, such as brain-derived neurotrophic factor (BDNF), which promote neuroplasticity and improve neural connectivity (9, 18). Furthermore, regular physical activity has been associated with improvements in memory, attention, and executive function (19). These cognitive enhancements are attributed to exercise's capacity to increase cerebral blood flow, reduce inflammation, and promote cardiovascular health, all of which contribute to overall brain health (20).

Long COVID, also known as post-acute sequelae of SARS-CoV-2 infection (PASC), is a multisystem condition affecting a substantial proportion of COVID-19 survivors, characterized by persistent or newly emerging symptoms that appear weeks to months after the acute infection (21). The condition commonly manifests as profound fatigue, dyspnea, exercise intolerance, and markedly reduced functional capacity, with underlying mechanisms including systemic inflammation, skeletal muscle atrophy, metabolic and mitochondrial dysfunction, and, in some cases, autonomic dysregulation (22, 23). Research demonstrates that individuals with persistent long COVID symptoms have higher odds of physical inactivity compared to those without persistent symptoms, creating a concerning bidirectional relationship in which reduced physical activity may further exacerbate the condition's debilitating effects on exercise tolerance and overall functional capacity (14).

Cognitive dysfunction in post-COVID-19 patients includes difficulties with memory, attention, and executive functions. Studies suggest that brain inflammation and alterations in cerebral blood flow resulting from the immune response to the virus may contribute to these cognitive deficits (24, 25). The strong correlation between VO₂ max and cognitive performance measures observed in our study supports the hypothesis that physical fitness may mitigate these effects, potentially by reducing inflammation and enhancing endothelial function (36).

Physical exercise upregulates neurotrophic and plasticity pathways; increases in BDNF, GDNF, NGF, and their receptor expression accompany exercise protocols and are associated with improvements in learning and memory, as demonstrated in both human and animal studies (26). Exercise influences synaptic plasticity, hippocampal neurogenesis, epigenetic regulation, and neurotransmitter modulation; these processes are proposed to remodel neural circuits involved in memory encoding, consolidation, and retrieval (26, 27).

Graham et al. reported that 85% of long COVID patients experienced cognitive problems up to seven months after infection, consistent with our findings of significant cognitive dysfunction in post-COVID-19 patients. Additionally, Becker et al. found that deficits in processing speed, executive function, and memory were common among COVID-19 survivors, particularly those who had been hospitalized.

Specifically regarding VO₂, its relationship with cognitive performance represents a growing area of research with significant implications for understanding how aerobic fitness influences brain function. The most well-established mechanism linking VO₂ max to cognitive performance involves hippocampal plasticity. Exercise and aerobic fitness promote neurogenesis in the dentate gyrus, particularly benefiting hippocampal-dependent memory processes such as pattern separation and relational memory (18).

Our study complements these findings by demonstrating that physical fitness, as measured by VO_2_ max, is associated with improved cognitive outcomes (28, 29). The results of this study suggest that incorporating physical exercise programs into rehabilitation protocols for post-COVID-19 patients may be an effective strategy to mitigate cognitive deficits associated with long COVID. Furthermore, public policies promoting physical activity could benefit cognitive and mental health, thereby reducing the burden of long COVID symptoms. Regular physical exercise may not only enhance overall physical health but also provide significant cognitive benefits, contributing to the full recovery of post-COVID-19 patients (30).

Exercise-based rehabilitation has emerged as a promising therapeutic intervention for managing long COVID symptoms, with systematic reviews and meta-analyses consistently demonstrating clinically meaningful improvements across multiple outcomes. Studies indicate that structured physical activity programs, particularly those combining aerobic and resistance training, can lead to substantial gains, including 14%–15% increases in peak oxygen consumption (VO_2_ peak), 16%–33% improvements in muscle strength, and significant reductions in dyspnea and fatigue scores (15, 31).

Although the cross-sectional design limits causal inferences, we employed several strategies to enhance the robustness of the results, including adjustments for potential confounders such as age, sex, education level, BMI, disease severity, and comorbidities. Despite the relatively small and homogeneous sample, drawn from a single hospital center, it is highly representative of the studied population. However, it is necessary to acknowledge that these factors limit the generalizability of the results to broader populations of patients with long COVID. Thus, although the findings demonstrate internal consistency, they should be interpreted with caution, reinforcing the need for multicenter studies with larger samples to confirm and expand the understanding of the observed results. Furthermore, baseline cognitive performance and pre-infection physical fitness should also be considered, as they may have influenced the findings. Individuals with greater cognitive reserve or better physical fitness prior to SARS-CoV-2 infection may exhibit greater resilience to cognitive decline. Additionally, residual confounding factors, including socioeconomic status, pre-COVID physical activity levels, and comorbidities not fully captured by the Charlson Comorbidity Index, cannot be completely ruled out. These aspects should be considered when interpreting the results. Future research should investigate the causal relationship between physical fitness and cognitive function in post-COVID-19 patients. Larger, multicenter studies may enhance the validity of the findings, while exploring the underlying biological mechanisms could clarify how exercise influences cognition. Additionally, the use of neuroimaging techniques may provide valuable insights into brain changes associated with physical activity in this context.

Current evidence-based recommendations emphasize the importance of individualized, supervised, and progressive rehabilitation approaches for most long COVID patients, with careful attention to safety considerations and potential contraindications (32, 33). Systematic reviews report improvements in cognitive impairment when exercise is incorporated into multidisciplinary rehabilitation programs targeting post-COVID cognitive complaints (34). Correlative data from cohort studies link reductions in long COVID symptom burden with improvements in executive function over follow-up, suggesting that interventions—including physical activity—that reduce symptom severity may be accompanied by gains in executive functioning (35).

This study demonstrated a significant positive correlation between physical fitness, measured by VO_2_ max, and cognitive performance, assessed by the ACE-R, in patients who had been hospitalized for COVID-19. Our findings are consistent with existing literature suggesting that physical exercise can enhance cognitive function through mechanisms such as increased production of neurotrophic factors, improved neural connectivity, and reduced inflammation. We observed that physical fitness may serve as a mitigating factor for cognitive deficits commonly associated with long COVID, supporting the hypothesis that physical activity plays a crucial role in the cognitive recovery of these patients.

## Data Availability

The raw data supporting the conclusions of this article will be made available by the authors, without undue reservation.
